# Optimization of Oleuropein Extraction from Olive Leaves and Its Protective Effect Against TBHP-Induced Oxidative Damage in HEK-293 Cells

**DOI:** 10.3390/foods15152582

**Published:** 2026-07-23

**Authors:** Bingshuang Li, Jingyu Chen, Haodong Cheng, Zhaobin Wang, Enxiang Zhang, Feng Kong, Qinghua Zeng

**Affiliations:** 1 Shandong Key Laboratory of Applied Technology for Protein and Peptide Drugs, School of Pharmaceutical Sciences and Food Engineering, Liaocheng University, Liaocheng 252059, China; 2 State Key Laboratory for Macromolecule Drugs and Large-Scale Manufacturing, School of Pharmaceutical Sciences and Food Engineering, Liaocheng University, Liaocheng 252059, China

**Keywords:** olive leaf, oleuropein, response surface methodology, HEK-293 cells, oxidative damage, protective effect

## Abstract

Olive leaves, a major byproduct of olive processing, are generated in large quantities annually yet suffer from inefficient utilization and low added value. In this study, response surface methodology (RSM) was employed to optimize the extraction conditions of oleuropein from olive leaves. Additionally, the antioxidant activity of purified oleuropein and its protective effect against tret-butyl hydroperoxide (TBHP)-induced oxidative damage in the human embryonic kidney 293 (HEK-293) cell line were investigated. The optimal extraction conditions for oleuropein were determined as follows: extraction temperature of 71 °C, extraction time of 72 min, ethanol concentration of 58%, and solid–liquid ratio of 1:27 (mg/mL), yielding an oleuropein recovery of 44.5%. The extract was purified and identified as oleuropein via Fourier transform infrared spectroscopy (FTIR) and high-performance liquid chromatography (HPLC). Oleuropein exhibited remarkable antioxidant activity and mitigated TBHP-induced oxidative damage in HEK-293 cells by inhibiting apoptosis. TBHP treatment reduced cell viability by approximately 70%, while treatment with 100 and 200 μg/mL oleuropein restored the decreased cell viability to 100%. Morphological observations and 4′,6-diamidino-2-phenylindole (DAPI) staining revealed that TBHP induced apoptotic cell death characterized by nuclear condensation and fragmentation, and this effect was reversed by oleuropein treatment. Flow cytometry analysis showed that TBHP caused approximately 90% cell death, whereas co-treatment with oleuropein reduced cell death to only about 10%. TBHP downregulated the expression of p53, and oleuropein reactivated its expression, highlighting the role of oleuropein in the recovery of the cellular antioxidant system. This study possibly indicated the protective mechanism of oleuropein against oxidative damage-related diseases and provides a theoretical basis for the development of olive leaves as a potential ingredient in functional foods.

## 1. Introduction

Escalating resource scarcity and the growing consumer preference for natural products have raised awareness of the eco-economic value of waste biomass. The extraction of bioactive components from agri-food industry byproducts has garnered extensive attention due to their diverse health-promoting properties [1]. Olive leaves are a primary byproduct of olive cultivation and olive oil processing, with the global olive industry producing over 1 million tons of olive leaves annually. However, most olive leaves are underutilized and often discarded or incinerated, resulting in severe resource waste and environmental pollution [2]. Olive leaves are rich in antioxidant compounds such as phenols and flavonoids, which can scavenge reactive oxygen free radicals and alleviate oxidative stress in the body. Recent studies have confirmed that olive leaves can serve as a sustainable source of phenolic compounds via alcohol-based extraction [3,4], indicating broad prospects for the valorization of olive leaf resources and the application of their high-value extracts in food and biomedicine fields.

Phenolic compounds in olive leaves, including oleuropein, hydroxytyrosol, and luteolin, are the core components responsible for their nutritional and biological activities [5,6]. Among these, oleuropein accounts for 40–50% of the total phenolic content. As a non-toxic phenolic terpenoid glycoside, oleuropein exerts protective and reparative effects on various organs, is easily absorbed by the human body, and has great potential for application in health products. Recent research has demonstrated that oleuropein promotes neurological recovery and inhibits gastric cancer cell proliferation [7]. The olive-rich Mediterranean diet is associated with a significantly reduced incidence of respiratory diseases, tumors, and other chronic illnesses, and these beneficial effects are closely linked to oleuropein in olive products [8].

Oxidative stress-induced reactive oxygen species (ROS) can damage key cellular components, leading to altered membrane fluidity and permeability, as well as DNA fragmentation [9,10,11]. Oxidative stress is a well-recognized contributor to kidney disease, triggering a spectrum of pathological conditions from acute kidney injury to chronic renal failure, often accompanied by inflammatory responses [12]. Contrast-induced nephropathy, a serious complication of diagnostic radiological procedures, is characterized by elevated renal oxidative stress, increased thiobarbituric acid-reactive substances, and reduced glutathione (GSH) levels [13]. Extensive efforts have been made to identify antioxidant molecules that can protect the kidney from such oxidative damage [14]. Olive fruit extracts have been shown to exert antioxidant effects against deltamethrin- and H_2_O_2_-induced hepatotoxicity and nephrotoxicity [15]. However, the antioxidant effect of olive leaf extracts against TBHP-induced oxidative damage in kidney cells remains poorly understood.

Existing studies have explored oleuropein extraction from olive leaves with standard parameter ranges. Ethanol–water mixtures (40–80%) are commonly used to balance extraction efficiency and purity, paired with a solid–liquid ratio of 1:10–1:30 (g:mL) to promote mass transfer. Conventional extraction operates at 40–70 °C for 1–3 h. Moderate heating facilitates oleuropein release, whereas excessive temperature or extended treatment degrades thermolabile oleuropein and reduces yield.

Ultrasonic and microwave-assisted extraction effectively improve the low efficiency of conventional methods by disrupting plant cells and enhancing oleuropein recovery. Although supercritical CO_2_ extraction yields high-purity, residue-free products, its industrial use is limited by high costs. Current research largely focuses on single-factor optimization, lacking systematic parameter synergism exploration and low-cost scalable green extraction strategies. Accordingly, this study optimizes oleuropein extraction conditions to develop a reliable, practical process for the high-value utilization of olive leaf by-products.

Efficient optimization of the extraction process is a critical step in promoting the development and utilization of oleuropein from olive leaves, a renewable resource. In this study, RSM was used to optimize the extraction conditions of oleuropein by investigating the effects of multiple extraction parameters on its yield. Furthermore, cell viability, morphological changes, antioxidant protein expression, and apoptotic levels were assessed to clarify the protective effect of purified oleuropein on TBHP-induced oxidative damage in HEK-293 cells. This study aims to provide a technical basis for the high-value utilization of olive leaf resources and reveal the potential of oleuropein as a natural antioxidant for the prevention and mitigation of oxidative stress-related kidney diseases.

## 2. Materials and Methods

Olive leaves sourced from Wudu District, Longnan City, Gansu Province, China, harvested on 17 February 2023. Botanical identification of the experimental olive (*Olea europaea* L.) plant material was strictly confirmed in this study. The fresh olive leaves used for oleuropein extraction were collected from artificially cultivated *Olea europaea* L. trees. Secondly, to ensure the accuracy of the experimental material, the collected olive leaf samples were further verified and identified by professional botanists from the local agricultural and forestry research institution. A voucher specimen of the olive leaf material has been preserved in the laboratory specimen repository of our research group for future reference and verification.

1,1-Diphenyl-2-picrylhydrazyl (DPPH), tert-butyl hydroperoxide (TBHP), dimethyl sulfoxide (DMSO), fetal bovine serum (FBS), 3-(4,5-dimethyl-2-thiazolyl)-2,5-diphenyl-2H-tetrazolium bromide (MTT), and oleuropein standard were purchased from Sigma-Aldrich (Shanghai, China). 2,2′-Azino-bis (3-ethylbenzothiazoline-6-sulfonic acid) diammonium salt (ABTS) was obtained from Solarbio (Beijing, China). The HEK-293 cell line was provided by the National Collection of Authenticated Cell Cultures (Shanghai, China). Dulbecco’s Modified Eagle’s Medium (DMEM), phosphate-buffered saline (PBS), and 4′,6-diamidino-2-phenylindole (DAPI) were purchased from Thermo Fisher Scientific (Shanghai, China). 2′,7′-Dichlorodihydrofluorescein diacetate (H_2_DCFDA) was obtained from Biofount (Beijing, China). The PE-Annexin V/7-AAD Cell Apoptosis Detection Kit was purchased from BD Biosciences (San Jose, CA, USA). All other reagents used were of analytical grade.

### 2.1. Optimization of Oleuropein Extraction

#### 2.1.1. Preliminary Extraction of Oleuropein from Olive Leaves

Dried olive leaves were ground and sieved through a 100-mesh sieve to obtain a fine powder. A 5 g sample of olive leaf powder was extracted with 60% ethanol at a solid–liquid ratio of 1:26 (mg/mL) in a water bath at 70 °C for 90 min. The mixture was centrifuged at 5000 r/min for 10 min, and the oleuropein content in the supernatant was determined by spectrophotometry at a wavelength of 282 nm [16].

#### 2.1.2. Single-Factor Experiments

Four key extraction factors were investigated via single-factor experiments: extraction time (40–140 min), extraction temperature (50–90 °C), solid–liquid ratio (1:14–1:30, mg/mL), and ethanol concentration (40–80%). The fixed parameters for the single-factor experiments were set as follows: extraction temperature 70 °C, extraction time 90 min, solid–liquid ratio 1:26 (mg/mL), and ethanol concentration 60%. Each experiment was performed in triplicate.

#### 2.1.3. RSM Experimental Design

Based on the results of the single-factor experiments, a Box–Behnken design (BBD) with four variables and three levels was adopted to optimize the oleuropein extraction conditions. The four independent variables were: A (extraction time, 65, 90, 115 min), B (extraction temperature, 60, 70, 80 °C), C (solid–liquid ratio, 22, 26, 30 mg/mL), and D (ethanol concentration, 50%, 60%, 70%). Design-Expert 13 software (Minneapolis, MN, USA) was used for experimental design, data fitting, and regression analysis. A second-order polynomial equation was established to describe the relationship between the dependent variable (oleuropein yield) and the independent variables, as shown in Equation (1):

(1) $$Y=\beta_{0}+\sum_{i=1}^{3}\beta_{i}X_{i}+\sum_{i=1}^{3}\beta_{ii}X_{i}^{2}+\sum_{i=1}^{3}\sum_{j=i+1}^{3}\beta_{ij}X_{i}X_{j}$$

where Y is the predicted oleuropein yield; Xi and Xj are the coded values of the independent variables; β0 is the intercept; βi, βii, and βij are the linear, quadratic, and interactive regression coefficients, respectively. Analysis of variance (ANOVA) was used to evaluate the significance of the regression model and each coefficient, with *p* < 0.05 considered statistically significant. The model’s adequacy was assessed based on the lack of fit, coefficient of determination (R^2^), and F-value. Three-dimensional (3D) response surface plots and contour plots were generated to visualize the interactive effects of the variables on oleuropein yield, and the optimal extraction conditions were predicted based on the model.

### 2.2. Purification of Oleuropein

The crude olive leaf extract was dissolved in 10% aqueous ethanol (*v*/*v*) to yield a 2 mg/mL solution. The sample was loaded onto a column packed with AB-8 macroporous resin (Solarbio Science & Technology, Beijing, China) and allowed to adsorb for 1 h. Oleuropein was then eluted with 70% ethanol (*v*/*v*) at a flow rate of 3 mL/min. The eluate was concentrated by rotary evaporation and freeze-dried to obtain purified oleuropein powder for subsequent experiments.

### 2.3. HPLC Identification of Oleuropein

Purified oleuropein (1 mg) was dissolved in 1 mL ethanol and filtered through a 0.22 μm organic phase filter membrane. HPLC analysis was performed on a Waters e2695-2998 chromatography (Milford, MA, USA) system equipped with a Venus II Prep G C18 column (Milford, MA, USA) (4.6 × 250 mm, 10 μm). The mobile phase consisted of H_2_O and acetonitrile (50:50, *v*/*v*) at a flow rate of 1.0 mL/min, with an injection volume of 10 μL [17]. The oleuropein standard was analyzed under the same conditions (282 nm, 25 °C, 10 min) for qualitative and quantitative identification.

### 2.4. FTIR Characterization of Oleuropein

A total of 5 mg of purified oleuropein was mixed with 150 mg of potassium bromide (KBr), ground into a fine powder, and pressed into a transparent pellet. FTIR spectra were recorded on a Nicolet iS50 spectrometer (Waltham, MA, USA) in the wave number range of 4000–400 cm^−1^. The functional groups of oleuropein were identified based on the characteristic absorption peaks in the FTIR spectrum (4 cm^−1^ in resolution, and 5 scans were applied for each spectrum).

### 2.5. Determination of Antioxidant Activity of Oleuropein

#### 2.5.1. DPPH Radical Scavenging Activity

The DPPH radical scavenging activity of oleuropein was determined with minor modifications [18]. Oleuropein was dissolved in 60% ethanol to prepare solutions with concentrations ranging from 0.4 to 1.0 mg/mL. A 0.5 mL aliquot of each oleuropein solution was mixed with 2.5 mL of 0.5 mmol/L DPPH ethanol solution, and the mixture was incubated in the dark at room temperature for 30 min. The absorbance was measured at 517 nm using a UV–visible spectrophotometer (Santa Clara, CA, USA). A 0.2 mg/mL vitamin C solution was used as the positive control, and 60% ethanol was used as the blank control.

#### 2.5.2. ABTS Radical Scavenging Activity

The ABTS radical scavenging activity was determined with slight modifications [18]. Oleuropein solutions (0.4–1.0 mg/mL) were prepared with 60% ethanol. A 0.05 mL aliquot of the sample solution was mixed with 4 mL of ABTS working solution, and the absorbance was measured at 734 nm after incubation at room temperature for 6 min. A 0.2 mg/mL vitamin C solution was used as the positive control, and 60% ethanol was used as the blank control. All experiments were performed in triplicate, and the radical scavenging rate was calculated according to the standard formula.

### 2.6. Protective Effect of Oleuropein on Hek-293 Cells

#### 2.6.1. Cell Culture and Treatment

HEK-293 cells were cultured in DMEM supplemented with 10% FBS and 1% penicillin–streptomycin, and maintained in a humidified incubator at 37 °C with 5% CO_2_. When the cell confluence reached 50–60% (24–48 h after seeding) [19], the medium was replaced, and the cells were pretreated with different concentrations of oleuropein for 2 h, followed by exposure to TBHP to induce oxidative damage. A blank control group (no treatment) and a TBHP model group (only TBHP treatment) were set up for comparison.

#### 2.6.2. Cell Viability Assay (MTT Method)

The protective effect of oleuropein on TBHP-induced cytotoxicity was evaluated via the MTT assay. After the above treatment, the culture medium was replaced with fresh medium containing 0.5 mg/mL MTT, and the cells were incubated at 37 °C for 4 h. The MTT solution was discarded, and 150 μL DMSO was added to each well to dissolve the formazan crystals. The absorbance was measured at 490 nm using a microplate reader, and the cell viability was calculated relative to the blank control group.

#### 2.6.3. Apoptosis Analysis by Flow Cytometry

Cell apoptosis was detected using the PE-Annexin V/7-AAD double staining method. Treated HEK-293 cells were harvested, washed twice with cold PBS, and resuspended in binding buffer at a concentration of 1 × 10^6^ cells/mL. Then, 5 μL PE-Annexin V and 10 μL 7-AAD were added to the cell suspension, mixed gently, and incubated in the dark at room temperature for 10 min. Apoptosis was analyzed within 30 min using a flow cytometer (Burlington, MA, USA), and the apoptotic rate was calculated using the corresponding analysis software.

#### 2.6.4. DAPI Staining for Nuclear Morphology Observation

HEK-293 cells were seeded on glass coverslips and treated as described above. The cells were fixed with methanol for 15 min at room temperature, washed twice with PBS, and stained with DAPI working solution for 15 min in the dark. After washing twice with methanol, the coverslips were mounted on glass slides, and nuclear morphology was observed under a laser confocal microscope. Apoptotic cells were identified by characteristic nuclear changes such as condensation, fragmentation, and punctation.

#### 2.6.5. Intracellular Ros Level Detection

Intracellular ROS levels were detected using the fluorescent probe H_2_DCFDA [17]. Treated cells were incubated with 10 μmol/L H_2_DCFDA at 37 °C for 30 min in the dark, washed three times with cold PBS to remove excess probe, and then observed under a laser confocal microscope. The green fluorescence intensity was used to reflect the intracellular ROS level, with higher fluorescence intensity indicating higher ROS production. Three groups were set up: blank control group, TBHP model group, and TBHP + oleuropein co-treatment group. And Image J (versin: 1.8.0) was used to quantify the intensity of ROS figures.

#### 2.6.6. Western Blot Analysis of Antioxidant and Apoptotic Proteins [10]

Treated cells were lysed with ice-cold RIPA lysis buffer containing a protease inhibitor cocktail, and the total protein concentration was determined using the BCA protein assay kit. Equal amounts of protein (30 μg per lane) were separated by sodium dodecyl sulfate-polyacrylamide gel electrophoresis (SDS-PAGE) and transferred to PVDF membranes from SEVENBIO (Bejing, China) with Cat: SW220-02. The membranes were blocked with 5% skim milk for 1 h at room temperature, then incubated with primary antibodies against Bcl-2 (1:1000, cat: 15071, CST), Bax (1:1000, cat: 2772, CST), p53 (1:1000, cat: 2524, CST), caspase3 (1:1000, cat: 9662, CST) and β-actin (1:10,000, cat: 4970, CST) at 4 °C overnight. After washing three times with TBST, the membranes were incubated with horseradish peroxidase (HRP)-conjugated secondary antibodies: anti-mouse IgG HRP-linked antibody (Thermo Fisher, 31430) and anti-rabbit IgG HRP-linked antibody (Thermo Fisher, 31460) for 1 h at room temperature. The protein bands were visualized using an enhanced chemiluminescence (ECL) kit (AP34L014,Life-iLab, Shanghai, China).

### 2.7. Statistical Analysis [20]

All experimental data were expressed as the mean ± standard error (SE). Statistical analysis was performed using GraphPad Prism 9 software. Student’s *t*-test was used for comparison between two groups, and two-way ANOVA was used for comparison among multiple groups, followed by Tukey’s post hoc test. A *p*-value < 0.05 was considered statistically significant. Each experiment was repeated at least three times independently.

## 3. Results and Discussion

### 3.1. Effects of Single Factors on Oleuropein Yield

**Figure 1:**
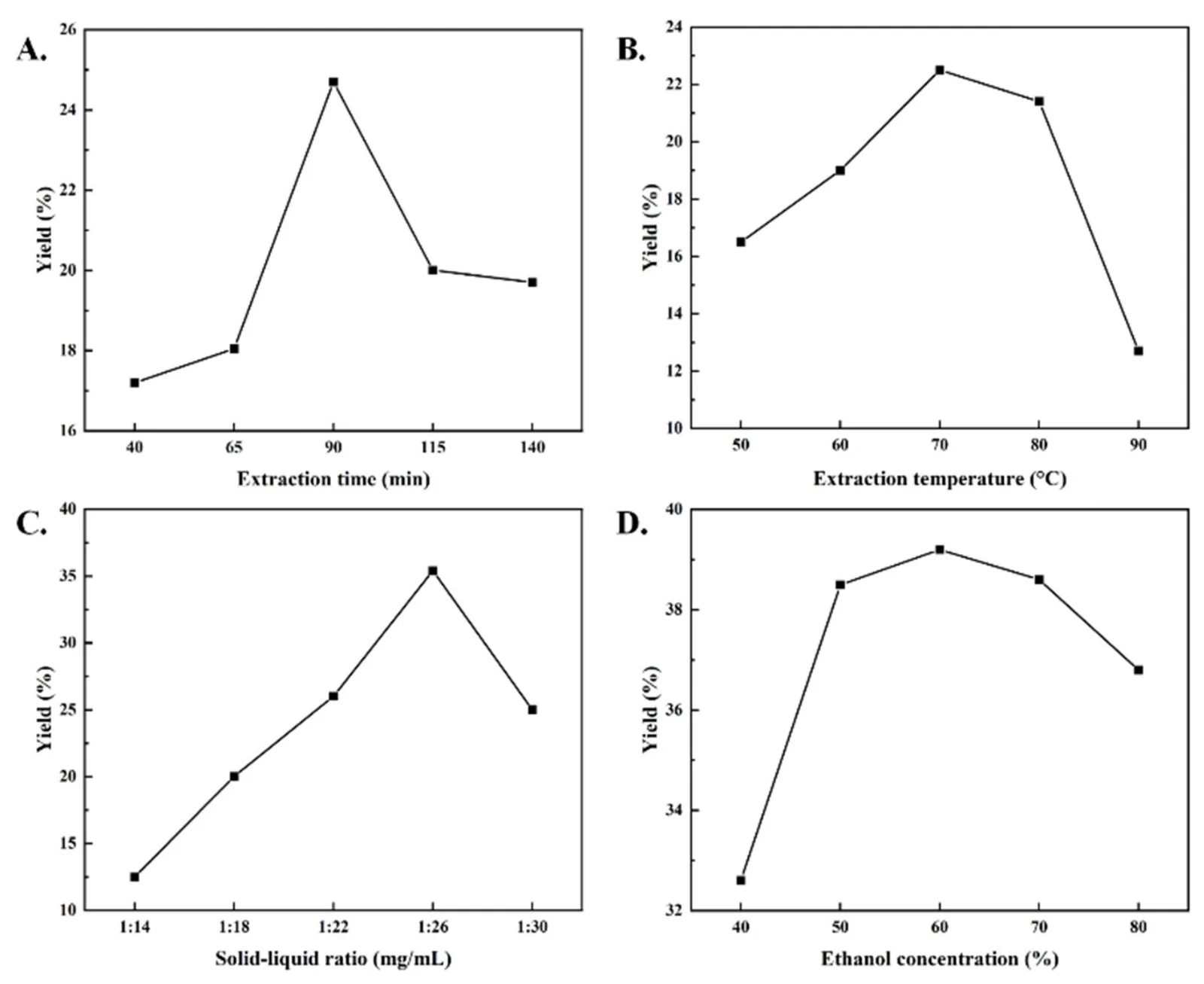
The effects of extraction time, temperature, solid–liquid ratio and ethanol concentration on the yield of oleuropein (**A**–**D**).

Single-factor experiments were conducted to investigate the effects of extraction time, temperature, solid–liquid ratio, and ethanol concentration on oleuropein yield, and the results are shown in Figure 1. As shown in Figure 1A, with the extension of extraction time from 40 to 140 min, the oleuropein yield first increased and then decreased, reaching a maximum of 24.70% at 90 min. Prolonging the extraction time beyond 90 min led to a decrease in yield, which may be attributed to the thermal degradation of oleuropein under prolonged heating conditions [21].

The effect of extraction temperature on yield (Figure 1B) showed a similar trend: the yield increased with temperature from 50 to 70 °C (maximum 22.50% at 70 °C) and then decreased at temperatures above 70 °C. Moderate temperature elevation accelerates molecular motion and mass transfer, promoting the dissolution of oleuropein from olive leaf matrix, while excessive temperature causes the degradation of oleuropein and reduces its yield [22].

As shown in Figure 1C, the oleuropein yield increased with the increase in solid–liquid ratio from 1:14 to 1:26 (mg/mL), peaking at 35.40% at 1:26, and then slightly decreased at a higher solid–liquid ratio (1:30). An appropriate increase in solvent volume can enhance the mass transfer driving force and promote the release of oleuropein, but excessive solvent volume leads to dilution of the extract and increased difficulty in subsequent purification, without a significant increase in yield [23]. Ethanol concentration had a significant effect on oleuropein yield (Figure 1D): the yield increased with ethanol concentration from 40% to 60% (maximum at 60%) and then decreased at concentrations above 60%. This phenomenon may be explained by two reasons: first, oleuropein has limited solubility in high-concentration ethanol; second, oleuropein has a wide polarity range, and excessively high ethanol concentration reduces the polarity of the solvent system, leading to a decrease in the solubility of oleuropein and thus a lower extraction yield [24]. The results of the single-factor experiments provided a reasonable range of variables for the subsequent RSM optimization.

### 3.2. RSM Modeling and Optimization of Extraction Conditions

**Figure 2:**
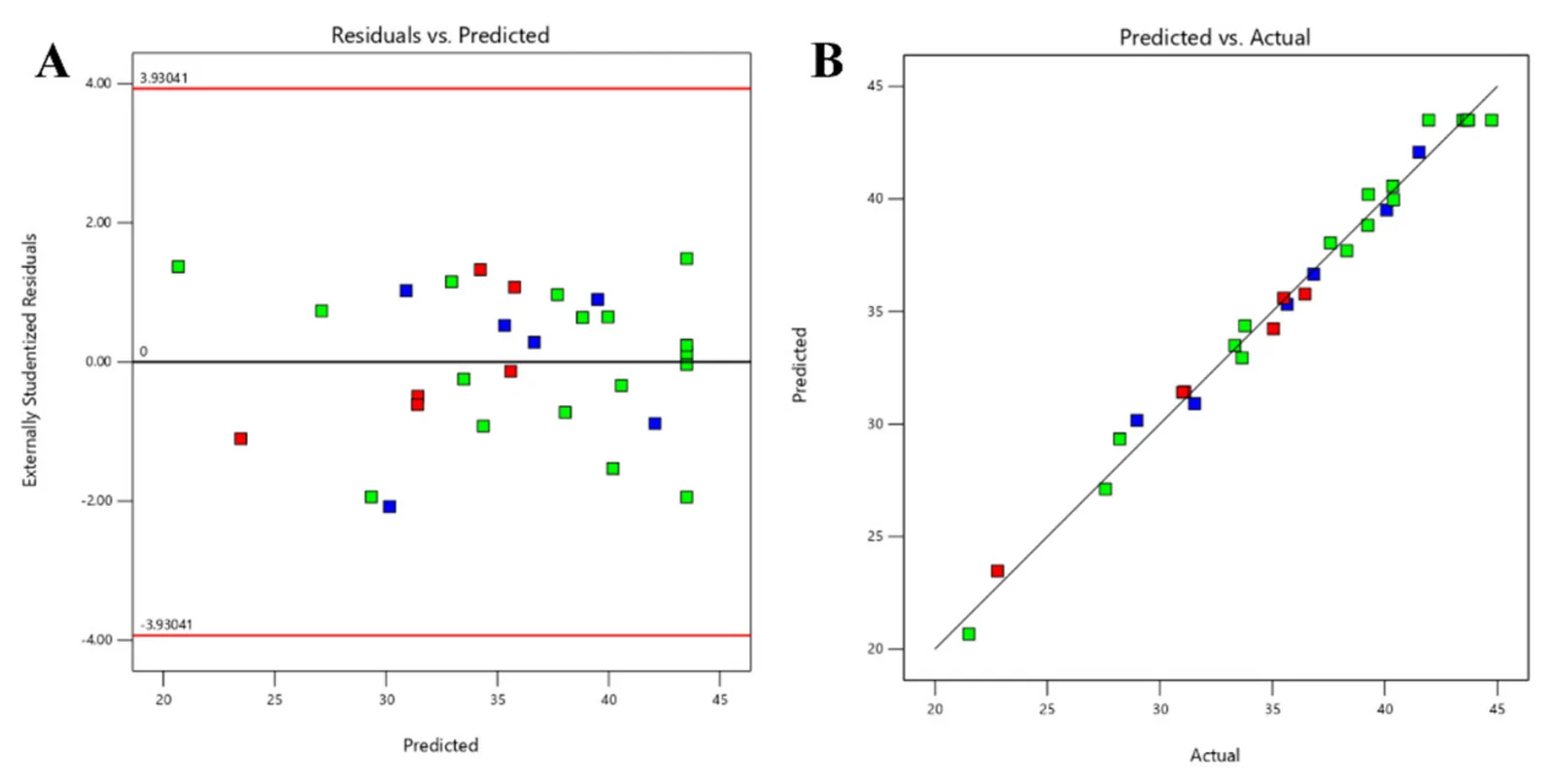
The studentized residuals versus the predicted yield (**A**) and regression plot of predicated value versus actual data of yield (**B**).

RSM is an efficient statistical method for optimizing multi-factor processes, which can reduce the number of experiments and accurately reveal the interactive effects of variables [25]. Based on the single-factor experiment results, a BBD with four variables and three levels was designed, and 29 experimental runs were performed (Table S1). The adequacy of the regression model was first evaluated via residual analysis: the studentized residuals of oleuropein yield were randomly distributed around the zero line within the range of ±3.93 (Figure 2A), indicating that the experimental data had constant variance and no outliers, and the model was suitable for data fitting without additional transformation [26]. The plot of actual yield vs. predicted yield (Figure 2B) showed that all data points were closely distributed around the diagonal line, indicating a high degree of agreement between the model-predicted values and the actual experimental values, and the model had good predictive ability. Compared with the published research, we got the yield of 44.5% total polyphenol yield, which is higher [27].

ANOVA was used to analyze the significance of the second-order polynomial regression model for oleuropein yield, and the regression equation (coded values) was obtained as follows:

Y = 43.5 − 2.03A − 2.15B + 4.7C − 1.89D − 3.28AB + 1.16AC + 1.35AD + 3.99BC + 2.15BD + 1.45CD − 1.61A2 − 5.1B2 − 6.9C2 − 5.09D2

where Y is oleuropein yield (%), A is extraction time, B is extraction temperature, C is solid–liquid ratio, and D is ethanol concentration.

The ANOVA results (Table 1) showed that the model had an F-value of 77.49 and *p* < 0.0001, indicating that the regression model was extremely significant. The lack of fit had an F-value of 0.9304 and *p* = 0.5812, which was not significant, suggesting that the model had no significant lack of fit and could well describe the relationship between the four variables and oleuropein yield. The coefficient of determination R^2^ was 0.9873, and the adjusted coefficient of determination Radj2 was 0.9745, both close to 1, indicating that 98.73% of the variation in oleuropein yield could be explained by the four independent variables, and the model had high fitting accuracy. The interactive terms (AB, AC, AD, BC, BD, CD) and all quadratic terms (A2, B2, C2, D2) were significant (*p* < 0.05), indicating that the variables had significant interactive and quadratic effects on oleuropein yield. Based on the F-values of the linear terms, the order of influence of the four variables on oleuropein yield was: solid–liquid ratio (C) > extraction temperature (B) > extraction time (A) > ethanol concentration (D).

**Figure 3:**
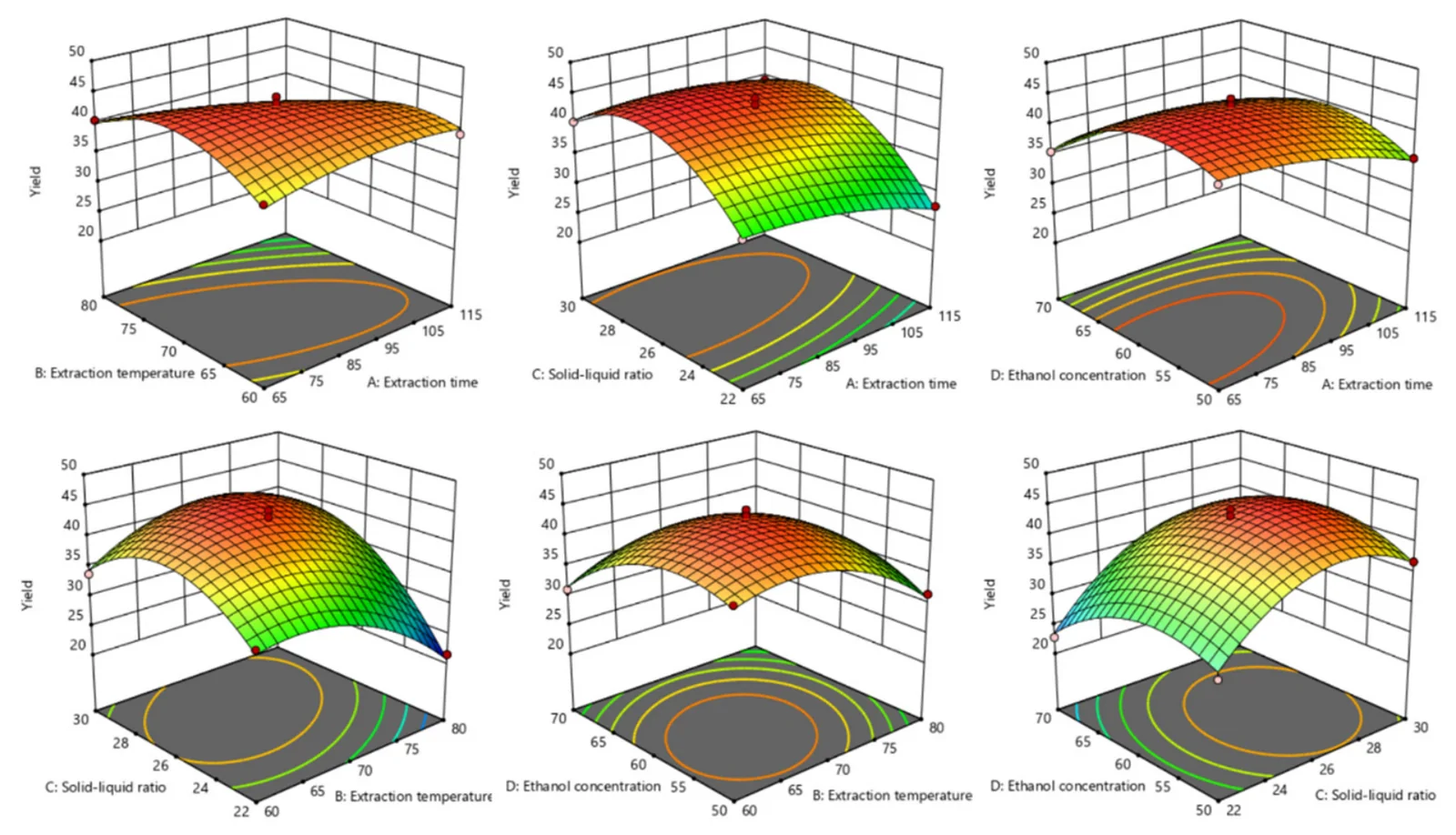
The three-dimensional (3D) response surface plots of oleuropein extraction on time, temperature, solid–liquid ratio and ethanol concentration for extraction yield as a function of significant interaction factors for RSM.

3D response surface plots and contour plots were generated to visualize the interactive effects of the variables on oleuropein yield (Figure 3). The interaction between extraction temperature and time (AB) was significant: the oleuropein yield increased with temperature up to 70 °C, and the effect of time on yield was mild within the optimal temperature range. The interaction between solid–liquid ratio and extraction time (AC) showed a positive correlation with yield, i.e., the yield increased with the increase in solid–liquid ratio and appropriate extension of time. The interaction between extraction time and ethanol concentration (AD) also had a positive effect on yield within the experimental range. The interaction between extraction temperature and solid–liquid ratio (BC) was the most significant, with the oleuropein yield varying from 21.51% to 44.75% with the change in these two variables, indicating that the combination of moderate temperature and optimal solid–liquid ratio was the key to improving yield. The interaction between ethanol concentration and temperature (BD) showed that the yield increased with the increase in ethanol concentration from 50% to 70% and temperature from 60 °C to 80 °C. The interaction between ethanol concentration and solid–liquid ratio (CD) showed that the maximum yield was obtained at 60% ethanol concentration and a solid–liquid ratio of 1:26 (mg/mL), which was consistent with the single-factor experiment results.

Numerical optimization was performed via Design-Expert 13 software to maximize the oleuropein yield, and the optimal extraction conditions were predicted as follows: extraction temperature 70.79 °C, extraction time 72.37 min, ethanol concentration 57.77%, and solid–liquid ratio 1:27.13 (mg/mL), with a predicted oleuropein yield of 45.00%. Considering the actual experimental operation, the conditions were adjusted to a more practical set: extraction temperature 71 °C, extraction time 72 min, ethanol concentration 58%, and solid–liquid ratio 1:27 (mg/mL). Three validation experiments were conducted under these optimized conditions, and the actual average oleuropein yield was 44.5%, which was consistent with the model-predicted value (relative error < 1.1%), indicating that the regression model was reliable and the optimized extraction conditions were practical and effective.

### 3.3. Structural Characterization of Purified Oleuropein

**Figure 4:**
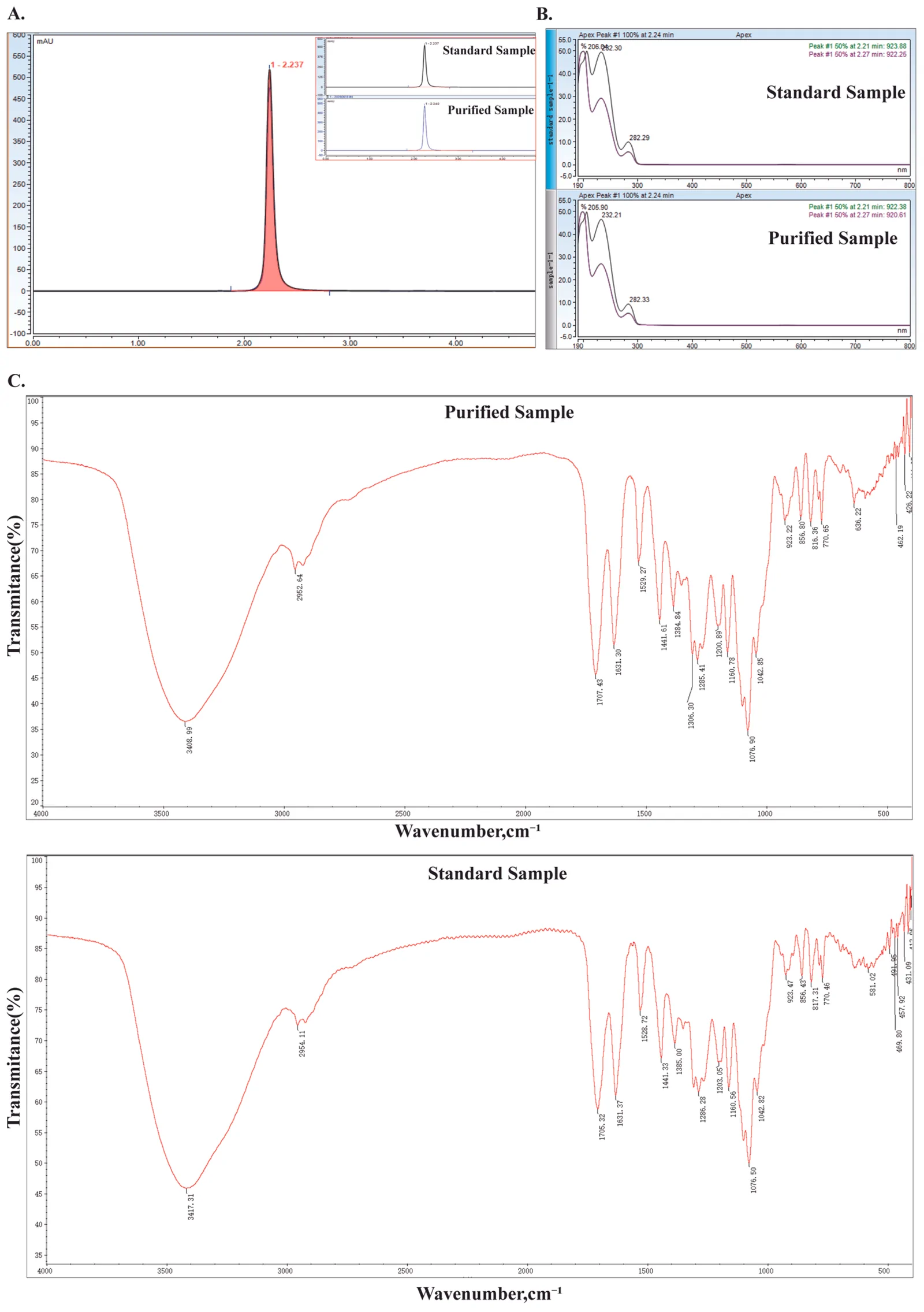
HPLC and FTIR of oleuropein ((**A**) HPLC; (**B**) UV–Vis absorption spectrum; (**C**) FTIR).

HPLC and FTIR were used to identify the purified olive leaf extract, and the results are shown in Figure 4. The HPLC chromatogram (Figure 4A) showed a single sharp peak at a retention time of 2.845 min, which was consistent with the retention time of the oleuropein standard, indicating that the purified extract had high purity and was identified as oleuropein [24]. The FTIR spectrum of the purified extract (Figure 4C) showed characteristic absorption peaks of oleuropein: a broad absorption band at 3417 cm^−1^ (3408 cm^−1^ of purified) was attributed to the O– H stretching vibration of aliphatic and phenolic hydroxyl groups, which is a typical characteristic of oleuropein; a peak at 2954 cm^−1^ (2952 cm^−1^ of purified)was assigned to the C-H stretching vibration of methyl and methylene groups; characteristic peaks at 1705, 1631, and 1528 cm^−1^ (1707, 1631, and 1529 cm^−1^ of purified)were due to C=O stretching, C=C stretching, and aromatic skeleton stretching, respectively [21]; a peak at 1441 cm^−1^ (1441 cm^−1^ of purified)was assigned to CH_2_ bending vibration; peaks at 1286cm^−1^ (1285 cm^−1^ of purified) and 1076 cm^−1^ (1076 cm^−1^ of purified) were attributed to C–O stretching vibration and C–O–C stretching vibration, respectively. The FTIR results confirmed that the purified extract contained secoiridoid structures with functional groups such as benzene ring, alkene, ether, and ester, which are the typical structural characteristics of oleuropein [24]. Combined with the HPLC results and UV–Vis absorption spectrum shown in Figure 4B, it was confirmed that the purified extract from olive leaves was oleuropein with high purity.

### 3.4. Antioxidant Activity of Oleuropein

**Figure 5:**
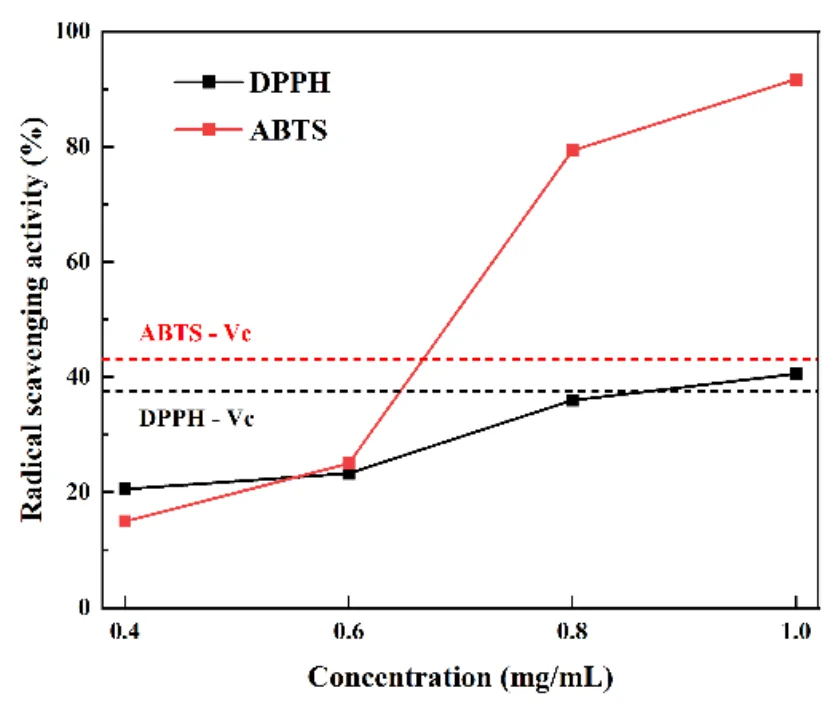
DPPH and ABTS radical scavenging activities of oleuropein solutions.

The DPPH and ABTS radical scavenging activities of oleuropein at different concentrations (0.4–1.0 mg/mL) were determined, with vitamin C (0.2 mg/mL) as the positive control, and the results are shown in Figure 5. The ABTS and DPPH radical scavenging rates of vitamin C were 43.32% and 37.66%, respectively. Both the DPPH and ABTS radical scavenging activities of oleuropein showed a significant concentration-dependent increase: the scavenging rates increased with the increase in oleuropein concentration in the range of 0.4–1.0 mg/mL. When the oleuropein concentration reached approximately 0.7 mg/mL, its ABTS radical scavenging activity was comparable to that of vitamin C; when the concentration reached approximately 0.9 mg/mL, its DPPH radical scavenging activity was close to that of vitamin C [28]. These results indicated that oleuropein had strong free radical scavenging ability and excellent *in vitro* antioxidant activity, which was consistent with previous studies showing that oleuropein is a natural antioxidant with high activity [22].

**Figure 6:**
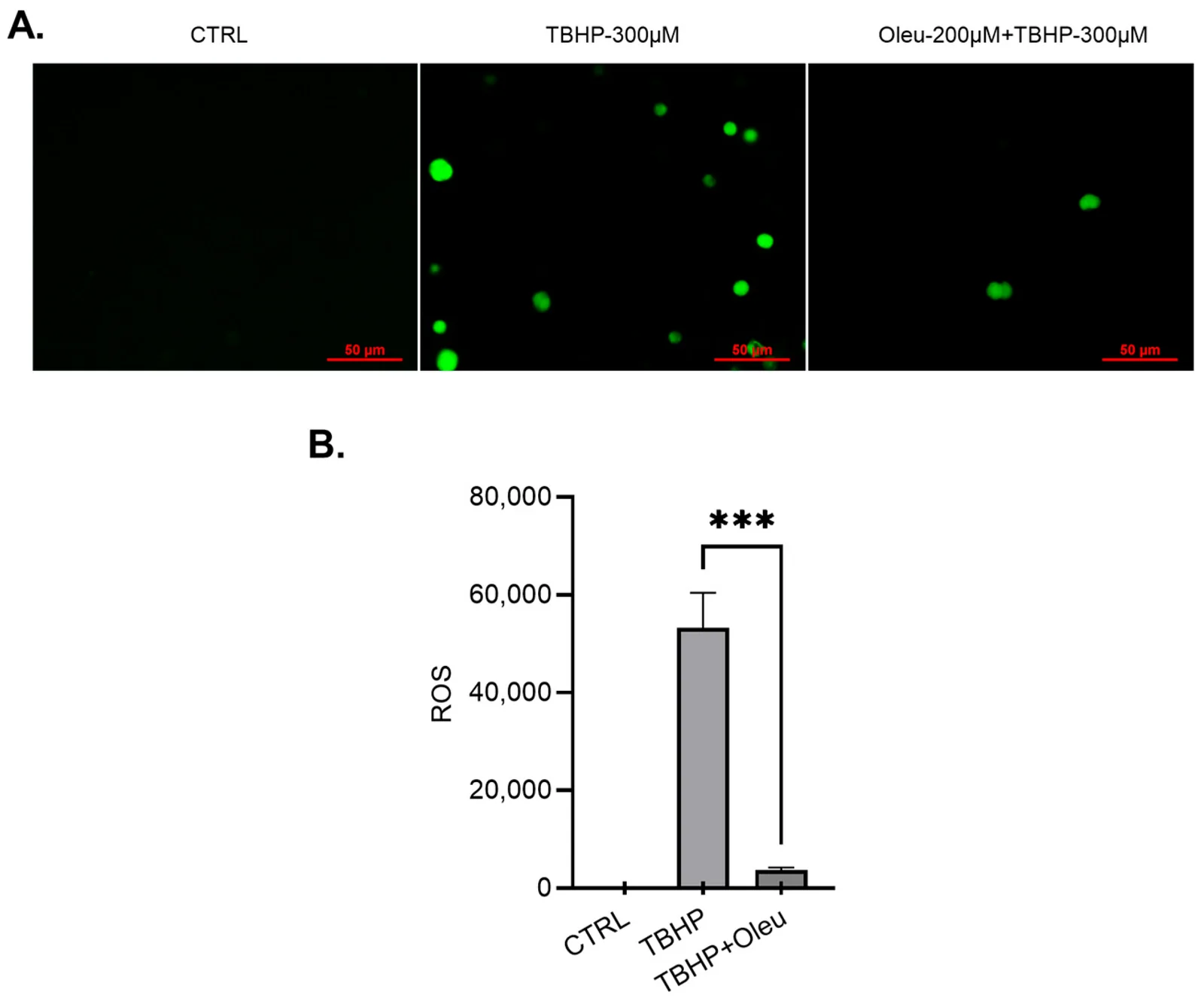
Effects of oleuropein on intracellular activation of oxygen in HEK-293 cells (**A**) Fluorescence picture of ROS induction; (**B**) Fluorescence intensity. *** *p* < 0.05.

Intracellular ROS levels were detected using the fluorescent probe H_2_DCFDA to evaluate the intracellular antioxidant activity of oleuropein, and the results are shown in Figure 6. H_2_DCFDA is a non-fluorescent probe that is oxidized to fluorescent 2′,7′-dichlorofluorescein (DCF) in the presence of ROS, and the green fluorescence intensity reflects the intracellular ROS level [17]. The blank control group showed weak green fluorescence (low ROS level), while the TBHP model group showed strong green fluorescence (Figure 6A), indicating that TBHP induced a significant increase in intracellular ROS production in HEK-293 cells, leading to severe oxidative stress [29]. In contrast, the TBHP + oleuropein co-treatment group showed significantly reduced green fluorescence intensity (Figure 6B), which was close to the blank control group, indicating that oleuropein could effectively scavenge intracellular ROS produced by TBHP and alleviate oxidative stress in HEK-293 cells. ROS is an important mediator of oxidative stress-induced cell damage, and the scavenging of intracellular ROS is the key mechanism by which oleuropein exerts its antioxidant effect.

### 3.5. Protective Effect of Oleuropein on Tbhp-Induced Apoptotic Damage in HEK-293 Cells

**Figure 7:**
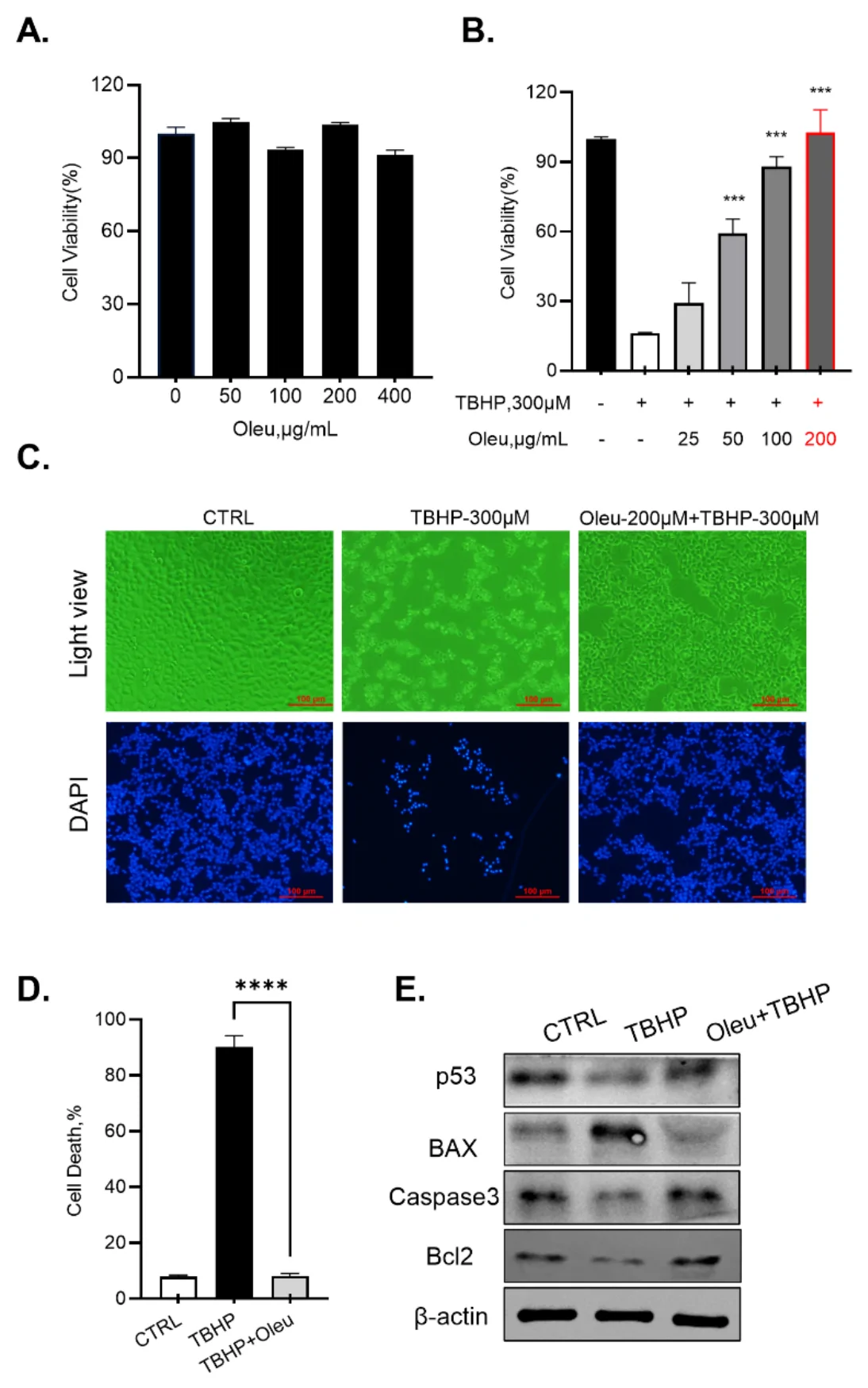
Oleuropein protects HEK-293 cells from apoptotic cytotoxicity induced by TBHP exposure (**A**) Effect of oleuropein on cell viability of HEK-293 cells; (**B**) Effect of oleuropein on TBHP-induced cell viability of HEK-293 cells; (**C**) Morphological observation of cell lines; (**D**) Detection of HEK-293 cells apoptosis by DAPI; (**E**) Western blot analysis of the protective role of oleuropein-200 μM). *** *p* < 0.05, **** *p* < 0.001.

Based on the excellent *in vitro* and intracellular antioxidant activity of oleuropein, its protective effect on TBHP-induced oxidative damage and apoptosis in HEK-293 cells was further investigated. First, the cytotoxicity of oleuropein on normal HEK-293 cells was evaluated via the MTT assay (Figure 7A). The results showed that oleuropein at concentrations ranging from 0 to 400 μg/mL had no significant effect on the viability of HEK-293 cells (*p* > 0.05), indicating that oleuropein has good biocompatibility and no cytotoxicity to normal kidney cells within this concentration range, which provides a basis for its subsequent application in cell protection experiments.

The protective effect of oleuropein on TBHP-induced cytotoxicity was then evaluated (Figure 7B). TBHP treatment alone reduced the viability of HEK-293 cells by approximately 70% compared with the blank control group, which was consistent with previous studies showing that oxidative stress induced by peroxides such as H_2_O_2_ and TBHP significantly reduces the viability of human kidney cells [30]. However, pretreatment with oleuropein significantly restored the cell viability in a concentration-dependent manner: the cell viability was restored to nearly 100% when the oleuropein concentration was 100 and 200 μg/mL, indicating that oleuropein could effectively protect HEK-293 cells from TBHP-induced cytotoxicity.

Morphological observations and DAPI staining were used to evaluate the effect of oleuropein on TBHP-induced nuclear damage (Figure 7C). The blank control group showed normal cell morphology with intact, uniformly stained nuclei; the TBHP model group showed obvious cell shrinkage, and DAPI staining revealed typical apoptotic nuclear changes such as condensation, fragmentation, and punctation, indicating that TBHP induced apoptotic cell death in HEK-293 cells. In contrast, the oleuropein co-treatment group showed normal cell morphology, and the nuclei were intact with uniform staining, without obvious condensation or fragmentation, indicating that oleuropein could effectively inhibit TBHP-induced nuclear damage and cell apoptosis.

Flow cytometry was used to quantitatively analyze the apoptotic rate of HEK-293 cells (Figure 7D), and the results showed that TBHP treatment induced approximately 90% cell death (including early and late apoptosis), while co-treatment with oleuropein reduced the cell death rate to only about 10%, which was close to the blank control group. This result further confirmed that oleuropein could significantly inhibit TBHP-induced apoptosis in HEK-293 cells, which was consistent with the morphological and cell viability results.

To explore the molecular mechanism of oleuropein’s protective effect, Western blot was used to detect the expression of p53 (Figure 7E). While p53 is widely recognized as a pivotal tumor suppressor that activates pro-apoptotic pathways under severe cellular stress, it also plays a nuanced role in modulating baseline antioxidant defenses and cellular homeostasis [31,32,33]. The results showed that TBHP treatment significantly downregulated the expression of p53 in HEK-293 cells, whereas oleuropein co-treatment restored p53 expression close to control levels. This suggests that oleuropein stabilizes homeostatic p53 expression to assist in regulating the oxidative stress response and mitigate the massive apoptotic cell death induced by severe chemical damage. In addition, downstream pro-apoptotic signaling activated by TBHP was suppressed by oleuropein, further confirming its cytoprotective effects. Collectively, these results demonstrated that oleuropein exerts a protective effect on TBHP-induced oxidative damage in HEK-293 cells by scavenging intracellular ROS, restoring the expression of antioxidant proteins, and inhibiting apoptotic cell death.

## 4. Conclusions

In this study, RSM was successfully used to optimize the extraction conditions of oleuropein from olive leaves, and the optimal conditions were determined as extraction temperature 71 °C, extraction time 72 min, ethanol concentration 58%, and solid–liquid ratio 1:27 (mg/mL), with an actual oleuropein yield of 44.5%. The purified extract was identified as oleuropein with high purity via HPLC and FTIR. Oleuropein exhibited excellent *in vitro* antioxidant activity (DPPH and ABTS radical scavenging) and intracellular antioxidant activity, which could effectively scavenge TBHP-induced intracellular ROS overproduction and alleviate oxidative stress in HEK-293 cells. Furthermore, oleuropein had no cytotoxicity to normal HEK-293 cells and could effectively protect the cells from TBHP-induced cytotoxicity and apoptotic damage: it restored the TBHP-decreased cell viability to 100%, reversed the apoptotic nuclear morphology, and reduced the cell death rate from 90% to 10%.

The molecular mechanism of this protective effect is related to the homeostatic stabilization of the regulatory protein p53 by oleuropein, which supports the recovery of the cellular antioxidant defenses and suppresses downstream apoptotic pathways activated by severe chemical oxidative stress.

This study provides a simple and efficient technical method for the high-yield extraction of oleuropein from olive leaves, realizing the valorization of olive leaf waste resources. Meanwhile, it suggested the potentially protective effect and mechanism of oleuropein against oxidative damage in kidney cells, providing a theoretical basis for the development of oleuropein as a natural antioxidant for the prevention and mitigation of oxidative stress-related kidney diseases [34].

## 5. Research Limitation

However, this study only provides experimental support for the application of olive leaves and oleuropein as potential ingredients in functional foods and health products *in vivo*. Future research can focus on the *in vivo* protective effect of oleuropein on oxidative damage-related kidney diseases and its industrial application technology to further expand the application scope of olive leaf resources and oleuropein. Additionally, those inner mechanisms were lacked. In the future, RNA seq application with animal model should be done to clarify the more concrete mechanisms.

## Figures and Tables

**Table 1:** Regression coefficient and ANOVA results of the quadratic polynomial model between response variable and independent variables.

Source	Sum of Squares	Degree of Freedom	Mean Square	F Value	*p* Value	
Model	1038.36	14	74.17	77.49	<0.0001	*
A	49.53	1	49.53	51.75	<0.0001	
B	55.34	1	55.34	57.82	<0.0001	
C	265.08	1	265.08	276.96	<0.0001	
D	42.98	1	42.98	44.90	<0.0001	
AB	43.03	1	43.03	44.96	<0.0001	
AC	5.38	1	5.38	5.62	0.0326	
AD	7.29	1	7.29	7.62	0.0153	
BC	63.60	1	63.60	66.45	<0.0001	
BD	18.58	1	18.58	19.41	0.0006	
CD	8.44	1	8.44	8.82	0.0101	
A^2^	16.71	1	16.71	17.46	0.0009	
B^2^	168.64	1	168.64	176.20	<0.0001	
C^2^	308.61	1	308.61	322.44	<0.0001	
D^2^	167.98	1	167.98	175.51	<0.0001	
Residual	13.40	14	0.9571			
Lack of Fit	9.37	10	0.9371	0.9304	0.5812	NS
Pure Error	4.03	4	1.01			
Cor Total	1051.76	28				
R^2^	0.9873					

*: significant; NS: not significant.

## Data Availability

The original contributions presented in this study are included in the Article and Supplementary Material. Further inquiries can be directed to the corresponding authors.

## Supplementary Materials

The following supporting information can be downloaded at: https://www.mdpi.com/article/10.3390/foods15152582/s1, Figure S1: HPLC result of oleuropein (Upper, standard; Lower, purified oleuropein); Figure S2: HPLC result overlap of oleuropein standard and purified oleuropein; Figure S3: UV absorption spectrum result of oleuropein (Upper, standard; Lower, purified oleuropein); Figure S4: Peak purity index value of oleuropein standard and purified oleuropein(Upper, standard; Lower, purified oleuropein ); Figure S5: FTIR of oleuropein standard and purified oleuropein (Upper, standard; Lower, purified oleuropein); Figure S6: Detection of HEK-293 cell apoptosis by flow cytometry (A, control group; B, treated by 500 μM TBHP; C, co-treated by oleuropein and TBHP); Table S1: Box–Behnken design for the response values and independent variables with three process parameters and three coded levels for each parameter.
