# Correction: Li et al. Optimization of Oleuropein Extraction from Olive Leaves and Its Protective Effect Against TBHP-Induced Oxidative Damage in HEK-293 Cells. *Foods* 2026, *15*, 2582

**DOI:** 10.3390/foods15183218

**Published:** 2026-09-11

**Authors:** Bingshuang Li, Jingyu Chen, Haodong Cheng, Zhaobin Wang, Enxiang Zhang, Feng Kong, Qinghua Zeng

**Affiliations:** 1 Shandong Key Laboratory of Applied Technology for Protein and Peptide Drugs, School of Pharmaceutical Sciences and Food Engineering, Liaocheng University, Liaocheng 252059, China; 2 State Key Laboratory for Macromolecule Drugs and Large-Scale Manufacturing, School of Pharmaceutical Sciences and Food Engineering, Liaocheng University, Liaocheng 252059, China

## Text Correction

There was an error in the original publication [1]. We did not provide the information concerning the redissolving of the extract before purification on hydrophobic resin.

A correction has been made to the first paragraph of Subsection 2.2. Purification of Oleuropein in the 2. Materials and Methods. 

The crude olive leaf extract was dissolved in 10% aqueous ethanol (*v*/*v*) to yield a 2 mg/mL solution. The sample was loaded onto a column packed with AB-8 macroporous resin (Solarbio Science & Technology, Beijing, China) and allowed to adsorb for 1 h. Oleuropein was then eluted with 70% ethanol (*v*/*v*) at a flow rate of 3 mL/min. The eluate was concentrated by rotary evaporation and freeze-dried to obtain purified oleuropein powder for subsequent experiments.

The authors state that the scientific conclusions are unaffected. This correction was approved by the Academic Editor. The original publication has also been updated.
