# Supplementary material for: Optimization of Oleuropein Extraction from Olive Leaves and Its Protective Effect Against TBHP-Induced Oxidative Damage in HEK-293 Cells
Source: Foods. 2026 Jul 23;15(15):2582. doi: 10.3390/foods15152582 (PMC13465481; doi:10.3390/foods15152582)
Supplement: Supplementary file 1 [file foods-15-02582-s001.zip › foods-4331809-supplementary.pdf]

**Supplementary Figure S1：**

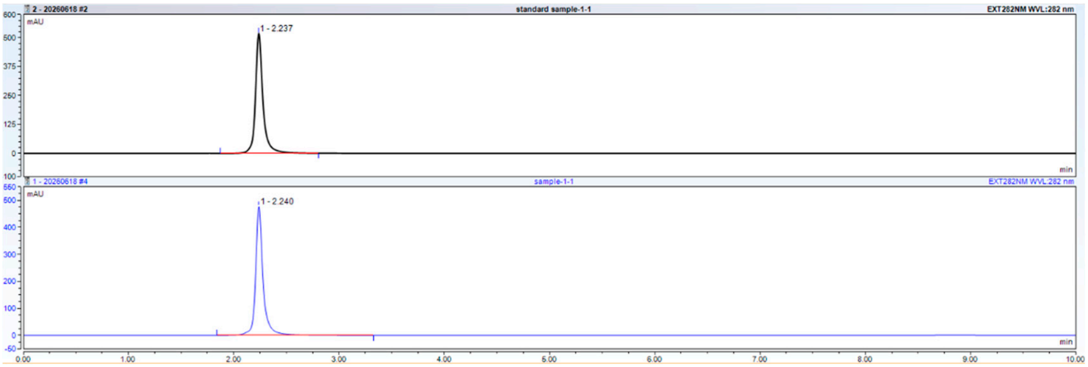


HPLC result of oleuropein (Upper, standard; Lower, purified oleuropein ).

**Supplementary Figure S2:**

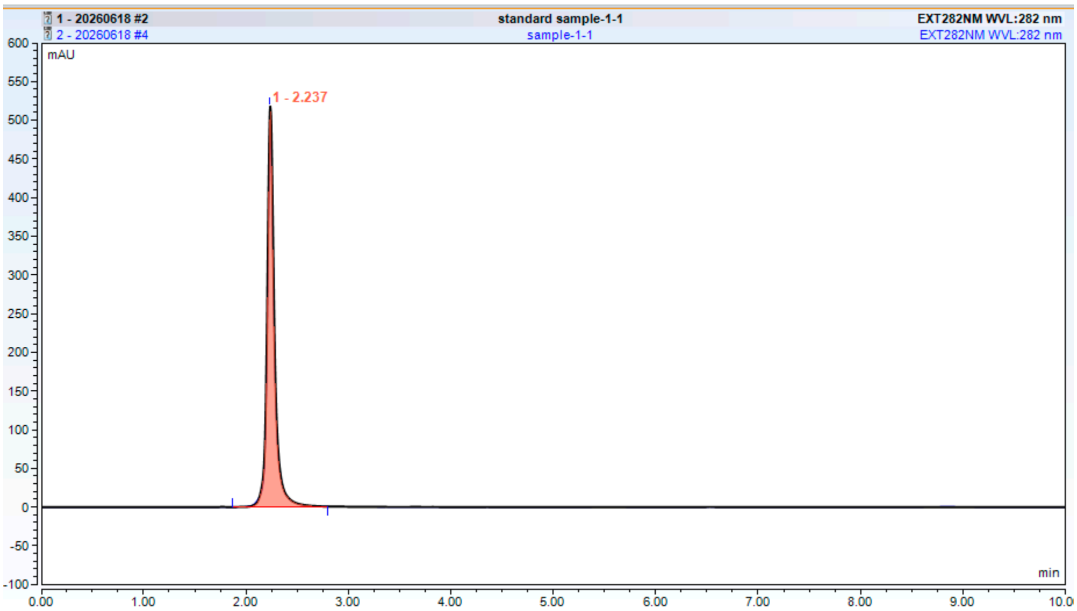


HPLC result overlap of oleuropein standard and purified oleuropein

**Supplementary Figure S3:**

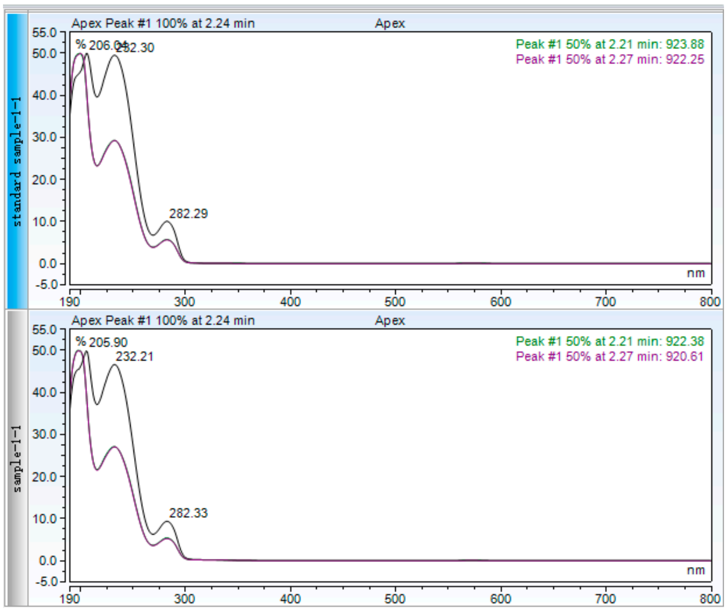


UV absorption spectrum result of oleuropein (Upper, standard; Lower, purified oleuropein ).

**Supplementary Figure S4:**

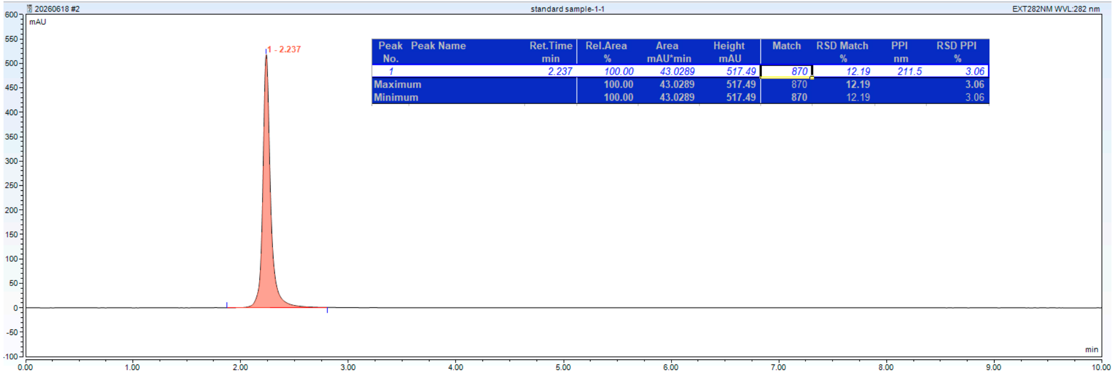


| Peak No. | Peak Name | Ret.Time min | Rel.Area % | Area mAU*min | Height mAU | Match | RSD Match % | PPI nm | RSD PPI % |
|---|---|---|---|---|---|---|---|---|---|
| 1 | | 2.237 | 100.00 | 43.0289 | 517.49 | 870 | 12.19 | 211.5 | 3.06 |
| Maximum | | | 100.00 | 43.0289 | 517.49 | 870 | 12.19 | | 3.06 |
| Minimum | | | 100.00 | 43.0289 | 517.49 | 870 | 12.19 | | 3.06 |

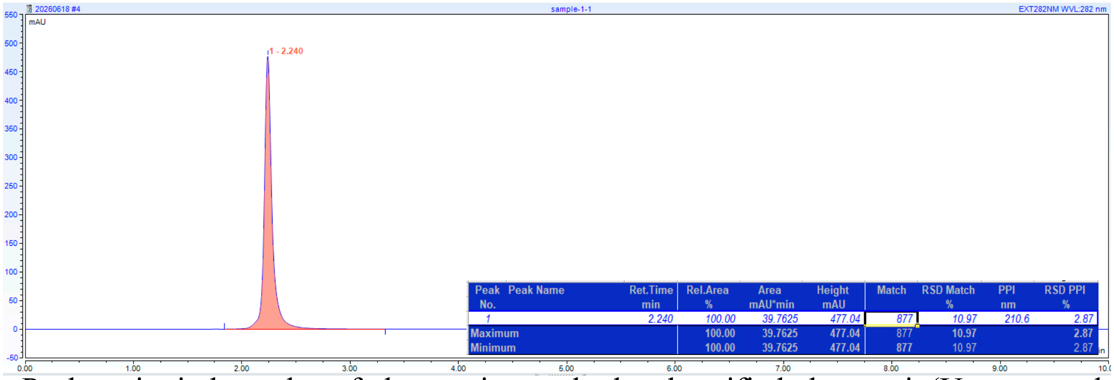


| Peak No. | Peak Name | Ret.Time min | Rel.Area % | Area mAU*min | Height mAU | Match | RSD Match % | PPI nm | RSD PPI % |
|---|---|---|---|---|---|---|---|---|---|
| 1 | | 2.240 | 100.00 | 39.7625 | 477.04 | 877 | 10.97 | 210.6 | 2.87 |
| Maximum | | | 100.00 | 39.7625 | 477.04 | 877 | 10.97 | | 2.87 |
| Minimum | | | 100.00 | 39.7625 | 477.04 | 877 | 10.97 | | 2.87 |

Peak purity index value of oleuropein standard and purified oleuropein(Upper, standard; Lower, purified oleuropein ).

**Supplementary Figure S5:**

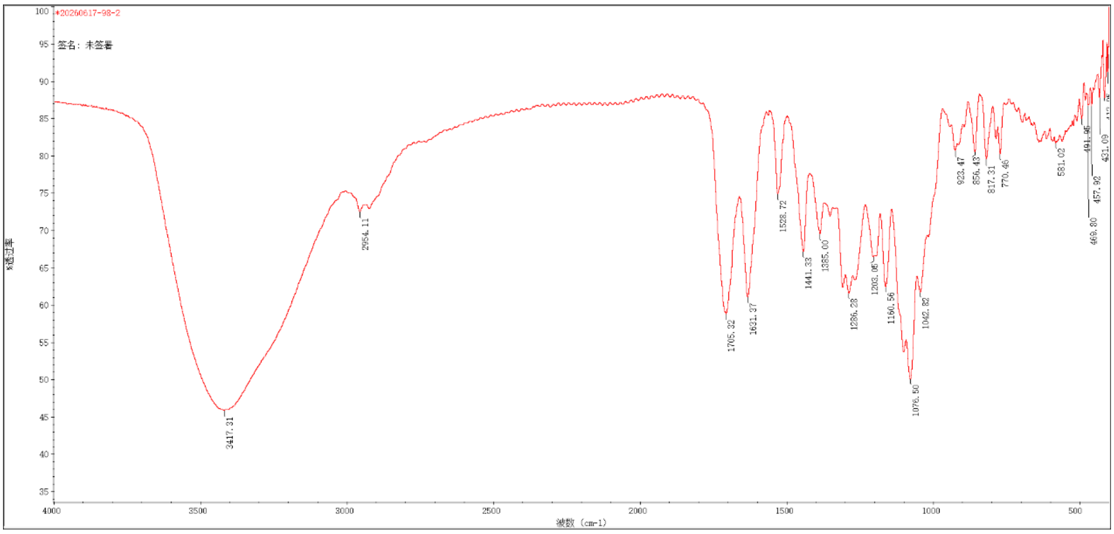


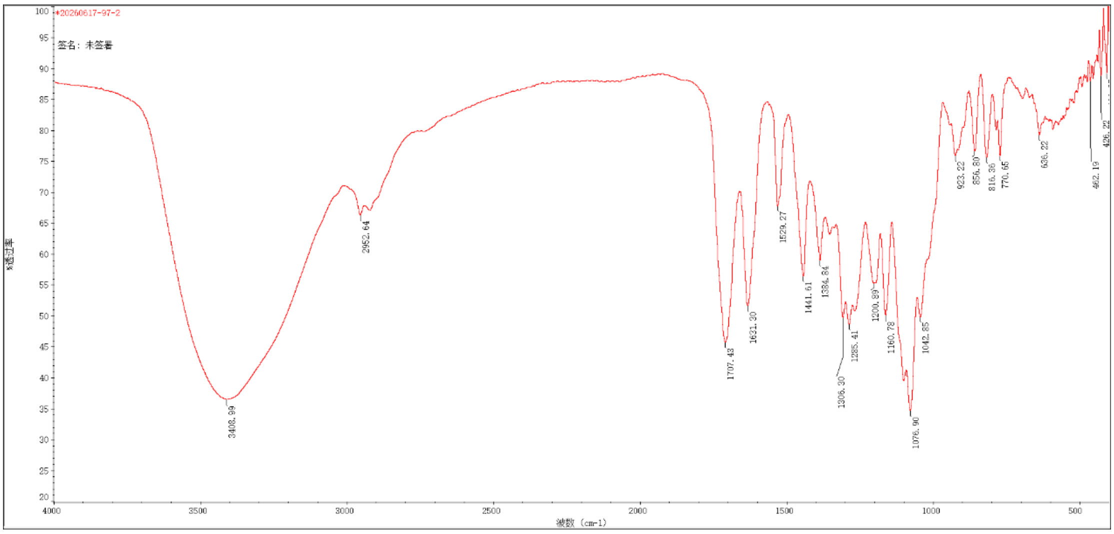


FTIR of oleuropein standard and purified oleuropein(Upper, standard; Lower, purified oleuropein ).

**Table S1.** Box-Behnken design for the response values and independent variables with three process parameters and three coded levels for each parameter.

| A-Time (min) | B-Temperature (°C) | C-Solid-liquid ratio (mg/mL) | D-Ethanol concentration (%) | Y-Yield (%) |
|---|---|---|---|---|
| 65 | 60 | 26 | 60 | 38.31 |
| 115 | 60 | 26 | 60 | 39.27 |
| 65 | 80 | 26 | 60 | 40.38 |
| 115 | 80 | 26 | 60 | 28.22 |
| 90 | 70 | 22 | 50 | 28.98 |
| 90 | 70 | 30 | 50 | 36.84 |
| 90 | 70 | 22 | 70 | 22.78 |
| 90 | 70 | 30 | 70 | 36.45 |
| 65 | 70 | 26 | 50 | 41.52 |
| 115 | 70 | 26 | 50 | 35.66 |
| 65 | 70 | 26 | 70 | 35.51 |
| 115 | 70 | 26 | 70 | 35.05 |
| 90 | 60 | 22 | 60 | 33.66 |
| 90 | 80 | 22 | 60 | 21.51 |
| 90 | 60 | 30 | 60 | 33.78 |
| 90 | 80 | 30 | 60 | 37.58 |
| 65 | 70 | 22 | 60 | 33.33 |
| 115 | 70 | 22 | 60 | 27.58 |
| 65 | 70 | 30 | 60 | 40.35 |
| 115 | 70 | 30 | 60 | 39.24 |
| 90 | 60 | 26 | 50 | 40.08 |
| 90 | 80 | 26 | 50 | 31.55 |
| 90 | 60 | 26 | 70 | 31.02 |
| 90 | 80 | 26 | 70 | 31.11 |
| 90 | 70 | 26 | 60 | 43.62 |
| 90 | 70 | 26 | 60 | 43.47 |
| 90 | 70 | 26 | 60 | 41.95 |
| 90 | 70 | 26 | 60 | 43.72 |
| 90 | 70 | 26 | 60 | 44.75 |

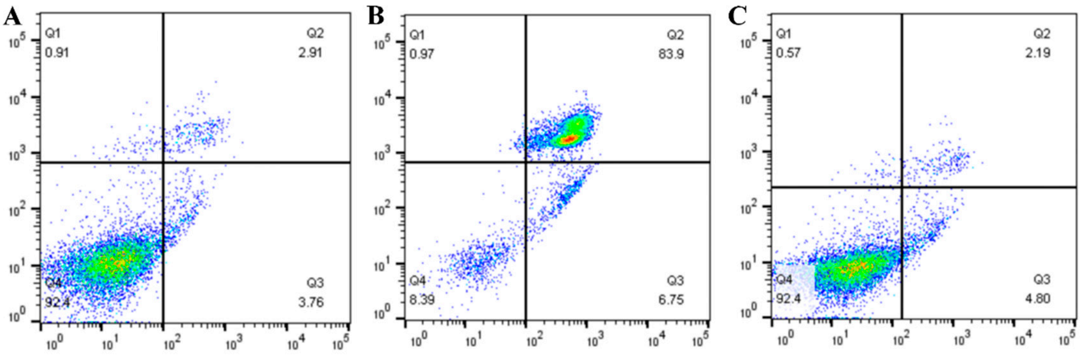


**Figure. S6** Detection of HEK-293 cell apoptosis by flow cytometry (A, control group; B, treated by 500 μM TBHP; C, co-treated by oleuropein and TBHP).
